# Regular physical activity moderates the adverse impact of type 2 diabetes on brain atrophy independently from HbA1c

**DOI:** 10.3389/fendo.2023.1135358

**Published:** 2023-02-17

**Authors:** Carolina Moreno, Otília C. d’Almeida, Leonor Gomes, Isabel Paiva, Miguel Castelo-Branco

**Affiliations:** ^1^ Faculty of Medicine, University of Coimbra, Coimbra, Portugal; ^2^ Department of Endocrinology, Coimbra University and Hospital Centre (CHUC), Coimbra, Portugal; ^3^ Coimbra Institute for Biomedical Imaging and Translational Research (CIBIT), Institute of Nuclear Sciences Applied to Health (ICNAS), University of Coimbra, Coimbra, Portugal

**Keywords:** type 2 diabetes, brain volume, MRI, cerebral atrophy, physical activity

## Abstract

**Objective:**

Brain atrophy has been consistently associated with type 2 diabetes, beginning in early stages of dysglycemia, independently from micro and macrovascular complications. On the contrary, physical activity relates with larger brain volumes. Our aim is to assess the influence of regular physical activity on brain volumes in people with type 2 diabetes.

**Methods:**

A cross-sectional multimodal evaluation with 3T MRI was performed on 170 individuals: 85 individuals with type 2 diabetes and 85 controls. They underwent clinical examination, blood sampling and 3T MRI. Brain volumes (mm^3^) were estimated using FreeSurfer 7. Physical activity duration was self-reported by the participants as the number of hours of physical activity per week for at least the previous 6 months. Statistical analysis was performed with IBM SPSS 27.

**Results:**

People with type 2 diabetes had significantly lower cortical and subcortical volumes, adjusted for age and individual intracranial volume, comparing to controls. Regression analysis showed that within type 2 diabetes group, lower gray matter volumes were associated with lesser physical activity duration (hours/week), independently from HbA1c. Moreover, there were significant moderate positive correlations between regular physical activity duration and gray matter volumes of cortical and subcortical subregions, specifically in the diabetes group.

**Conclusions:**

This study reveals a putative beneficial effect of regular physical activity independently of glycemic control, as assessed by HbA1c, which might contribute to reduce the negative impact of type 2 diabetes in the brain.

## 1 Introduction

Type 2 diabetes is associated with several brain structural abnormalities, mainly gray matter volume reduction, especially in older adults (1). These brain changes might be independent from microvascular and macrovascular complications, and precede neurological impairment (2). Alongside with vascular lesions (3), numerous mechanisms have been implicated in diabetes-associated neural injury, such as accumulation of advanced glycation end products (4), neuronal insulin resistance (5) and neuroinflammation (6). However, the impact of physical activity modulating these risk factors is not fully understood. Taking into consideration the ageing population and rapidly progressive prevalence of type 2 diabetes (7), it is imperative to identify the mechanisms underlying the links between diabetes and brain diseases and potential early interventions.

Several studies have investigated associations between physical activity and brain morphology. Despite some heterogeneity in findings, the majority suggested that physical activity relates with larger brain volumes (less brain atrophy) in older adults (8–10). Yet, there is a scarcity of data regarding the impact of regular physical activity on functional and structural brain health parameters of type 2 diabetes.

Review papers by Callisaya et al. and Bertram et al. highlight a possible effect of physical activity preventing or delaying dementia. The mechanisms hypothesized vary between improvement of endothelial function, attenuation of oxidative stress and increase of testosterone levels (11, 12). A systematic review by Podolski et al. with over 7000 individuals with type 2 diabetes concluded that physical activity could potentially contribute to improvement of cognitive performance (13). However, no link with brain morphometry was established.

The putative influence of physical activity on type 2 diabetes-related brain structural abnormalities is interesting, as it may precede cognitive decline and could play a key role in delaying neurodegenerative processes (14). In the present study, a cross-sectional case-control investigation was conducted in order to explore regional cerebral correlates, particularly the influence of regular physical activity on brain volumes of type 2 diabetes individuals.

## 2 Methods

### 2.1 Study design

This study was approved by the Ethics Commission of the Faculty of Medicine of the University of Coimbra and followed the tenets of the Declaration of Helsinki. Written informed consent was obtained from all participants after research procedures had been fully explained. Individuals with type 2 diabetes were recruited from the Endocrinology Department of Coimbra’s Hospital and University Center and a control group from the local community.

### 2.2 Eligibility criteria

All participants fulfilled the inclusion criteria: age between 45 and 75 years-old, type 2 diabetes diagnosis confirmed by 2019 WHO criteria (15) with determination of fasting glucose, HbA1c, absence of diabetes auto-antibodies (type 2 diabetes group) or exclusion of type 2 diabetes according the same criteria (control group). In all participants the absence of the following exclusion criteria was confirmed: history of neurological or psychiatric disease, dementia or cognitive impairment, active malignancy, inflammatory disease, chronic drug or alcohol dependence, severe visual impairment. Patients with previous cerebrovascular accident or other cortical vascular pathology were excluded, as well as participants with cognitive decline or with incomplete MRI protocol or low-quality criteria.

### 2.3 Clinical evaluation and laboratory assessments

Participants were submitted to a thorough clinical exam performed by a team of physicians, which included personal medical history, complete physical exam with ophthalmology assessment (retinal fundus photographs and optical coherencetomography) for diagnosis and characterization of micro/macrovascular complications (diabetic peripheral neuropathy defined using the Toronto Consensus Statement (16), previous history of peripheral artery occlusion or myocardial infarction). Blood and urine samples were collected to determine inclusion criteria and disease status and diabetic nephropathy staging (urinary albumin-to-creatinine ratio). Both clinical and laboratory assessments were performed on the same day of imaging acquisition.

Regular physical activity was considered as any moderate aerobic exercise with an intensity of at least 3 Metabolic Equivalent of Task (MET) performed for at least the previous 6 months. A MET is the resting metabolic rate, representing the amount of oxygen consumed while sitting at rest and is equal to 3.5mL per kg per minute or 1 kcal (4.2 kJ) per kg per hour. Only participants that fulfilled these criteria were eligible for further analysis. Physical activity duration was self-reported by the participants as the number of hours of physical activity per week.

### 2.4 Imaging procedures

All participants were submitted to a Magnetic Resonance Imaging scanning protocol on a 3T Tim Trio scanner (Siemens, Germany) equipped with a 12-channel birdcage head coil. A high-resolution T1-weighted anatomical image was acquired using a three-dimensional Magnetization Prepared Rapid Acquisition Gradient Echo sequence (repetition time/echo time/inversion time 2530/3.42/1100 ms; flip angle 7°; field of view 256×256 mm^2^; 176 slices with 1×1×1 mm^3^ voxel size, GRAPPA acceleration factor 2).

### 2.5 MRI data analysis

MR image processing was conducted using FreeSurfer version 7.0 (https://surfer.nmr.mgh.harvard.edu) software, following the standard “recon-all” stream to obtain global and regional cortical and subcortical GM volumes. Cortical regions were defined according to the Desikan-Killiany DKT40 atlas (17).

### 2.6 Statistical analysis

Global cortical and subcortical gray matter (GM) volumes were compared between individuals with type 2 diabetes and controls, using multivariate ANCOVA models adjusted for age and estimated total intracranial volume (eTIV) as confounding effects. Multiple regression analysis were carried out to evaluate the impact of weekly exercise hours on global (sub)cortical GM volumes and HbA1c in people with type 2 diabetes.

Correlational analysis were performed independently in each group to explore relationships between normalized (divided by eTIV×100) GM volumes of cortical and subcortical regions and the number of weekly physical activity hours, using bivariate Spearman correlation.

All statistical analysis were run in SPSS version 28 using two-tailed hypothesis testing with a 5% significance level.

## 3 Results

Of the 190 participants (93 with type 2 diabetes) that enrolled in the study, 170 participants fulfilled the eligibility criteria and were included for analysis: 85 individuals with type 2 diabetes, mean age 60 ± 8.0 years, 35 female; 85 control subjects, mean age 51 ± 8.8 years, 48 female. The baseline clinical and demographic characteristics are detailed in **Table 1**.

**Table 1:** Demographics, clinical and volumetric characteristics of the study subjects.

	Type 2 diabetes (n=85)	Controls (n=85)	Statistics
**Age (years)**	61 (14)	49 (12)	z=-6.6; p<0.001
**Male : Female**	50:35	37:48	p=0.065
**BMI (Kg/m^2^)**	29.6 ± 4.87 (n=84)	25.4 ± 3.27 (n=83)	t (145.4)=6.5; p<0.001
**HbA1c (%, mmol/mol)**	9.5 ± 2.35, 80 ± 2 (n=85)	5.4 ± 0.35, 36 ± 0.9 (n=71)	t (88.4)=15.8; p<0.001
**Fasting glucose (mmol/mL)**	9.1 (4.7)	5.4 (0.8)	z=-9.1; p<0.001
**Physical exercice (hours/week)**	2 (2)	3 (3)	z=-1.6; p=0.110
**Duration of disease (years)**	12 (13)	–	–
Micro/macrovascular complications (yes)	62 (72.9%)	–	–
**- Nephropathy** **- Retinopathy** **- Neuropathy** **- Coronary artery disease** **- Peripheral artery disease**	35 (41.2%) 23 (27.1%) 8 (9.4%) 7 (8.2%) 3 (3.5%)	– – – – –	– – – – –
**Insulin therapy (yes)**	62 (73%)	–	–
Other antidiabetic medication			
**- Metformin** **- DPPIV inhibitors** **- Sulfonylureas**	49 (57.6%) 29 (34.1%) 8 (9.4%)	- - -	- - -
**Cortical volume (cm^3^)**	410 ± 40.5	440 ± 49.6	t (161.6)=-4.6; p<0.001
**Sub-cortical volume (cm^3^)**	51 ± 4.54	56 ± 6.19	t (154.1)=-6.4; p<0.001
**eTIV (cm^3^)**	1400 ± 203	1400 ± 239	t (163.7)=-0.3; p=0.800

Data are presented as n (%), mean ± SD, or median (IQR), as appropriate. For group comparisons, Student’s t-test (continuous variables with normal distribution), Mann-Whitney U-tests (continuous variables without normal distribution), and Fisher exact test (qualitative variables) were performed.

BMI, body mass index; eTIV, estimated total intracranial volume.

ANCOVA analyses of global (sub)cortical GM volumes between type 2 diabetes and control groups, controlling for age and eTIV, revealed significant differences for each region (cortical: F(1,166)=11.3, p<0.001; subcortical: F(1,166)=22.7, p<0.001) with greater atrophy in the diabetes group, as expected.

From the original cohort, 69 subjects (34 with type 2 diabetes and 35 controls) fulfilled the regular physical activity threshold and were further analyzed. Multiple linear regression was applied to test if physical activity duration (hours/week) significantly predicted gray matter (sub)cortical volumes of individuals with type 2 diabetes who exercised regularly (n=34), independently from HbA1c. For cortical volumes, the overall regression was statistically significant (R^2^adjusted=23.8%, F(2,31)=6.1, p=0.006). Physical activity duration significantly predicted GM volumes (β=0.74, p=0.008, CI95% [0.20,1.28]), but not HbA1c (β=-0.22, p=0.196, CI95% [-0.57,0.12]). Similar findings were found for total subcortical GM volumes (R^2^adjusted=15.1%, F(2,31)=3.9, p=0.030; physical activity: β=0.11, p=0.027, CI95% [0.01,0.21]; HbA1c: β =-0.03, p=0.350 CI95% [-0.09,0.03]).

**Figure 1:**
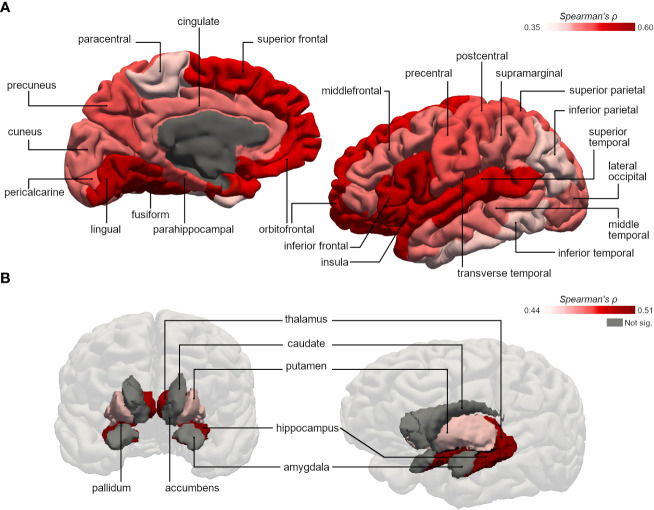
Representative 3D images of the parcellated and segmented brain regions assessed using FreeSurfer. Color intensity corresponds to the strength of the significant bivariate correlations between physical activity duration and gray matter volumes of **(A)** cortical areas (based on the DKT40 classifier atlas), and **(B)** subcortical areas, in type 2 diabetes group.

Physical activity duration was significantly correlated with gray matter volumes of several cortical and subcortical subregions in type 2 diabetes group. Regarding the cortex of individuals with type 2 diabetes, the correlations were more pronounced in frontal areas such as superior frontal (ρ=0.50, p=0.003), inferior frontal (ρ=0.55, p<0.001) and orbitofrontal gyrus (ρ=0.52, p=0.002); lingual (ρ=0.56, p<0.001) and fusiform gyrus (ρ=0.60, p<0.001) of the temporal lobe (**Figure 1A**). In the subcortical analysis, significant effects were identified in the hippocampus (ρ=0.51, p=0.002), thalamus (ρ=0.50, p=0.003), pallidum (ρ=0.48, p=0.004) and putamen (ρ=0.44, p=0.009) in which volumes were strongly correlated with physical activity duration (**Figure 1B**). Instead, in control group, physical activity duration showed no significant correlation with cortical or subcortical GM volumes aside from orbitofrontal gyrus (ρ=0.39, p=0.021). All results from correlation analysis are detailed in **Table 2**.

**Table 2:** Bivariate Spearman correlations between physical activity duration (hours/week) and regional (sub)cortical volumes normalized for eTIV (%) in type 2 diabetes and control groups.

Brain regions	Type 2 diabetes (n=34)	Controls (n=35)
Cortical regions		
** Superior frontal**	**ρ=0.50, p=0.003**	ρ=0.18, p=0.299
** Middle frontal gyrus**	**ρ=0.41, p=0.016**	ρ=0.15, p=0.385
** Inferior frontal gyrus**	**ρ=0.55, p<0.001**	ρ=0.03, p=0.858
** Orbitofrontal gyrus**	**ρ=0.52, p=0.002**	**ρ=0.39, p=0.021**
** Precentral gyrus**	**ρ=0.46, p=0.006**	ρ=0.01, p=0.935
** Paracentral lobule**	**ρ=0.38, p=0.027**	ρ=0.09, p=0.595
** Cuneus**	**ρ=0.42, p=0.012**	ρ=0.16, p=0.350
** Lateral occipital**	**ρ=0.40, p=0.018**	ρ=0.15, p=0.406
** Lingual**	**ρ=0.56, p<0.001**	ρ=0.14, p=0.413
** Pericalcarine**	**ρ=0.45, p=0.008**	ρ=0.11, p=0.537
** Superior parietal**	**ρ=0.48, p=0.004**	ρ=0.19, p=0.277
** Supramarginal**	**ρ=0.43, p=0.011**	ρ=-0.03, p=0.852
** Precuneus**	**ρ=0.45, p=0.008**	ρ=0.25, p=0.143
** Post central**	**ρ=0.46, p=0.007**	ρ=0.25, p=0.143
** Inferior parietal**	**ρ=0.36, p=0.037**	ρ=-0.03, p=0.879
** Entorhinal**	ρ=0.24, p=0.180	ρ=0.02, p=0.918
** Fusiform**	**ρ=0.60, p<0.001**	ρ=0.01, p=0.976
** Inferior temporal**	**ρ=0.39, p=0.022**	ρ=0.05, p=0.777
** Middle temporal**	**ρ=0.41, p=0.017**	ρ=0.13, p=0.456
** Parahippocampal**	**ρ=0.49, p=0.003**	ρ=-0.07, p=0.705
** Superior temporal**	**ρ=0.51, p=0.002**	ρ=0.07, p=0.703
** Transverse Temporal**	**ρ=0.42, p=0.013**	ρ=-0.07, p=0.708
** Insula**	**ρ=0.59, p<0.001**	ρ=0.16, p=0.348
** Cingulate**	**ρ=0.43, p=0.012**	ρ=0.18, p=0.301
Subcortical regions		
** Thalamus**	**ρ=0.50, p=0.003**	ρ=0.19, p=0.274
** Caudate**	ρ=0.30, p=0.081	ρ=-0.04, p=0.802
** Putamen**	**ρ=0.44, p=0.009**	ρ=0.02, p=0.909
** Pallidum**	**ρ=0.48, p=0.004**	ρ=-0.25, p=0.141
** Hippocampus**	**ρ=0.51, p=0.002**	ρ=-0.12, p=0.481
** Amygdala**	ρ=0.32, p=0.065	ρ=0.10, p=0.554
** Accumbens**	ρ=0.23, p=0.183	ρ=-0.06, p=0.718

Significant correlations are presented in bold.

## 4 Discussion

This study corroborates the commonly reported global gray matter volume reduction in type 2 diabetes versus controls (18, 19).To our knowledge this is the first study to assess a regional sensitivity of specific cortical and subcortical areas to physical activity in type 2 diabetes, revealing that physical activity contributes to a positive influence on gray matter integrity. Moreover, our findings suggests that this cortical and subcortical volume reduction might begin prior to cognitive decline. This outcome has implications for the understanding of pathophysiological mechanisms that promote structural brain changes in people with diabetes, as well as for the identification of modifiable factors that may play a role potentiating or decreasing brain atrophy. While some indisputable risk factors of diabetic brain changes have been reported: such as age (19), disease duration (20), micro and macrovascular complications (3, 21), or insulin resistance (22, 23) the impact of adjustable daily life aspects as nutrition or exercise maybe be positive, but not yet fully established (24).

Our results suggest that physical activity duration has an influence, amongst other factors, predicting brain volumes in type 2 diabetes, where individuals who exercised more regularly had larger (sub)cortical volumes. Surprisingly, this effect was independent from HbA1c, revealing possible beneficial outcomes of regular physical activity that surpasses the improvement of chronic mean glycemic control.

Physical activity duration seems to have a stronger correlation with the volume of specific brain regions, namely superior frontal, inferior frontal and orbitofrontal gyrus of the frontal lobe; lingual and fusiform gyrus of the temporal lobe; hippocampus, thalamus and pallidum. These are involved in executive function, decision and affective cognition as well as in perception, motor performance and memory (25). Ergo, the moderation of brain atrophy, will probably have a positive impact on several quality-of-life parameters of type 2 diabetes people who exercise regularly.

Previous works mainly addressed the influence of exercise on cognitive function and dementia, suggesting that certain types of physical activity appear to be beneficial to mental function in individuals with type 2 diabetes (12, 26). Zabetian-Targhi et al. found a positive correlation between hippocampal volume and attention-processing speed with step count, but not with global gray and white matter volumes (14). Other studies reported that hippocampal volume in older adults (20) and white matter hyperintensities (24) might also be influenced by regular physical activity in people with type 2 diabetes. Further studies, regarding the longitudinal impact of physical activity on detailed brain morphometry in a type 2 diabetes population need to be performed to confirm our results.

This study has some limitations regarding the quantification of physical activity, since this aspect was self-reported and there was no detailed information of specific aerobic and/or resistance training programs and performance outcomes. Also, the sample size of participants who engaged on regular physical activity was relatively small, revealing the sedentary habits of our study population (27).

In conclusion, the multifactorial therapeutic approach of type 2 diabetes must consider factors that might contribute to a preservation of structural brain integrity, such as physical activity, possibly preventing or delaying diabetes-related complications.

## Data availability statement

The raw data supporting the conclusions of this article will be made available by the authors, without undue reservation.

## Ethics statement

The studies involving human participants were reviewed and approved by Ethics Commission of the Faculty of Medicine of the University of Coimbra. The patients/participants provided their written informed consent to participate in this study.

## Author contributions

CM and OCdA processed the MR imaging data, carried out the statistical analysis and wrote the first draft of the manuscript. CM and LG selected participants and collected data. LG, IP and MC-B approved the revisions and final version of the manuscript. MC-B is responsible for the conception, funding and design of the study. MC-B is the guarantor of this work and, as such, had full access to all the data in the study and takes responsibility for the integrity of the data and the accuracy of the analysis. All authors contributed to the article and approved the submitted version.
